# A genome-wide trans-ethnic interaction study links the *PIGR-FCAMR* locus to coronary atherosclerosis via interactions between genetic variants and residential exposure to traffic

**DOI:** 10.1371/journal.pone.0173880

**Published:** 2017-03-29

**Authors:** Cavin K. Ward-Caviness, Lucas M. Neas, Colette Blach, Carol S. Haynes, Karen LaRocque-Abramson, Elizabeth Grass, Z. Elaine Dowdy, Robert B. Devlin, David Diaz-Sanchez, Wayne E. Cascio, Marie Lynn Miranda, Simon G. Gregory, Svati H. Shah, William E. Kraus, Elizabeth R. Hauser

**Affiliations:** 1 Duke Molecular Physiology Institute, Duke University School of Medicine, Durham, NC, United States of America; 2 Institute of Epidemiology II, Helmholtz Zentrum München, Neuherberg, Germany; 3 National Health and Environmental Effects Research Laboratory, US Environmental Protection Agency, Chapel Hill, NC, United States of America; 4 National Center for Geospatial Medicine, Rice University, Houston, TX, United States of America; 5 Division of Cardiology, Duke University School of Medicine, Durham, NC, United States of America; 6 Department of Biostatistics and Bioinformatics, Duke University School of Medicine, Durham, NC, United States of America; 7 Cooperative Studies Program Epidemiology Center-Durham, Veterans Affairs Medical Center, Durham, NC, United States of America; Universitatsklinikum Freiburg, GERMANY

## Abstract

Air pollution is a worldwide contributor to cardiovascular disease mortality and morbidity. Traffic-related air pollution is a widespread environmental exposure and is associated with multiple cardiovascular outcomes such as coronary atherosclerosis, peripheral arterial disease, and myocardial infarction. Despite the recognition of the importance of both genetic and environmental exposures to the pathogenesis of cardiovascular disease, studies of how these two contributors operate jointly are rare. We performed a genome-wide interaction study (GWIS) to examine gene-traffic exposure interactions associated with coronary atherosclerosis. Using race-stratified cohorts of 538 African-Americans (AA) and 1562 European-Americans (EA) from a cardiac catheterization cohort (CATHGEN), we identify gene-by-traffic exposure interactions associated with the number of significantly diseased coronary vessels as a measure of chronic atherosclerosis. We found five suggestive (P<1x10^-5^) interactions in the AA GWIS, of which two (rs1856746 and rs2791713) replicated in the EA cohort (P < 0.05). Both SNPs are in the *PIGR*-*FCAMR* locus and are eQTLs in lymphocytes. The protein products of both *PIGR* and *FCAMR* are implicated in inflammatory processes. In the EA GWIS, there were three suggestive interactions; none of these replicated in the AA GWIS. All three were intergenic; the most significant interaction was in a regulatory region associated with *SAMSN1*, a gene previously associated with atherosclerosis and B cell activation. In conclusion, we have uncovered several novel genes associated with coronary atherosclerosis in individuals chronically exposed to increased ambient concentrations of traffic air pollution. These genes point towards inflammatory pathways that may modify the effects of air pollution on cardiovascular disease risk.

## Introduction

Ischemic heart disease and stroke are responsible for more deaths worldwide than any other singular communicable or non-communicable cause [1]. The hallmark of ischemic heart disease coronary artery disease (CAD) is atherosclerotic lesions forming in the coronary arteries, eventually rupturing and releasing prothrombotic factors. The final result is an occlusive thrombus limiting blood flow and leading to a myocardial infarction [2]. Large scale genome-wide association studies have repeatedly identified genetic variants associated with CAD [3,4]. Though CAD is highly heritable, known genetic variants explain relatively little of the heritability, an observation possibly explained by as yet unreported interactions between genetic variants and environmental exposures [5].

Air pollution is a heterogeneous mixture of suspended particles and volatile organic and inorganic compounds, and the most widespread environmental exposure associated with CAD [6–8], with an estimated 22% of cardiovascular disease morbidity and mortality attributable to air pollution [9]. Air pollution is generated by a variety of industrial, agricultural, and residential/traffic sources. Vehicular traffic is one of the most common sources of air pollution; it is generated by tailpipe emissions, re-suspended road dust, and evaporative emissions. Exposure to traffic-related air pollution constituents and roadway noise is strongly associated with CAD [10–12]. The distance between the primary residence and nearest major roadway is a commonly used measure of traffic-related air pollution exposure that is closely correlated with air pollution emissions from traffic [13,14]. Distance to major roadways is associated with coronary heart disease [15–17], atherosclerosis [18], peripheral artery disease (PAD) [19], obesity [20], and metabolic risk factors for cardiovascular disease [21].

Gene-environment interactions may play a large role in cardiovascular disease [22]. However, studies to discover interactions between genes and air pollution have been mostly restricted to targeted studies of detoxification or inflammation-pathway associated genes [23]. While these candidate gene studies have been informative, they lack the comprehensive assessment available in genome-wide interaction studies (GWIS). A recent GWIS for gene-air pollution interactions identified genetic variants in the bone morphogenic protein gene family associated with PAD [24]. A smoking exposure GWIS identified several novel genetic variants associated with coronary artery calcification; these include genetic variants in *WWOX*, which are also associated with PAD via an interactions with smoking and traffic air pollution exposure [24,25].

To identify novel genes associated with coronary atherosclerosis in a traffic exposure GWIS, we used the CATHeterization GENetics (CATHGEN) cohort [26], which has proven to be a successful approach to study both cardiovascular disease and environmental exposures [21,24,27,28]. For our analyses we utilized a race-stratified study design, and used distance from the primary residence to the nearest major roadway as a measure of traffic-related air pollution exposure (traffic exposure).

## Materials and methods

### Study population

The CATHGEN cardiac catheterization cohort is composed of 9,334 individuals recruited through the Duke University cardiac catheterization laboratory from 2001–2011. Patients were administered a Health & Physical examination by a trained medical professional as part of the standard intake prior to the catheterization procedure. Demographic characteristics and peripheral blood were also collected prior to the procedure, and the patient’s Duke University medical records were linked to the CATHGEN database. A complete detailed description of CATHGEN is available [26]. Patient collection and data analyses associated with CATHGEN were approved by the Duke University Institutional Review Board.

**Fig 1:**
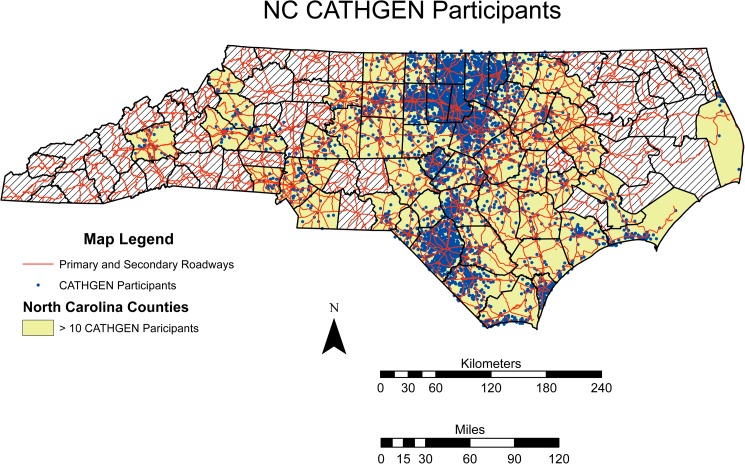
NC CATHGEN Participants. The distribution of CATHGEN participants overlaid with the primary and secondary roadway network (red lines) for North Carolina. To protect patient health information counties with 10 or fewer participants are suppressed and represented by hatch marks. All other counties have the CATHGEN participants locations (blue dots) randomized within a small area to preserve the overall structure and still protect patient health information.

Residential address information was available for 8,071 CATHGEN participants. To increase homogeneity, we restricted our study population to the 7,158 participants residing in North Carolina (**Fig 1**), of which 2,100 had genome-wide genotyping data. These 2,100 North Carolina residents with genome-wide genotyping comprised the study cohort.

### Clinical information

All clinical information was obtained from a pre-procedure interview and Duke University medical records incorporated into the CATHGEN database via the PEDIGENE^®^ system. Clinical variables were age, race, sex, smoking, BMI, presence of hypertension, hyperlipidemia, and type 2 diabetes (diabetes). Race was a self-reported variable used to stratify the cohort into self-identified African-Americans (AA) and European-Americans (EA). Hypertension, hyperlipidemia, and diabetes were all defined clinically by the operating team from pharmacologic treatment lists, and from clinical chemistries in the medical record or obtained prior to catheterization (**Table 1**). The coronary atherosclerosis outcome was defined as the number of diseased coronary vessels (number of diseased vessels, NUMDZV) at the catheterization. NUMDZV is a clinician annotated clinical variable that describes the number (0–3) of vessels with significant (> 75%) blockage while taking into account the location of the vessels as well as the left or right dominance of the patient. Disease in the left main carotid artery of greater than 50% is considered two-vessel disease, incrementing the NUMDZV variable by 2. Thus, NUMDZV is a measure of the degree of clinically significant coronary atherosclerosis.

**Table 1:** GWIS cohort demographic and clinical covariates.

GWIS Cohort Clinical Covariates	AA (N = 538)	EA (N = 1562)
Age (years)	56.3 (11.3)	61.2 (11.9)
BMI (kg/m^2^)	32.1 (8.01)	29.7 (7.02)
Distance to Roadways (km)	0.78 (0.71)	0.94 (0.81)
Sex (male)	288 (53.5)	619 (39.6)
Smoking (0 = never, 1 = ever)	231 (42.9)	789 (50.5)
Type 2 Diabetes (0 = no, 1 = yes)	232 (43.1)	415 (26.6)
Hypertension (0 = no, 1 = yes)	434 (80.7)	1008 (64.5)
Hyperlipidemia (0 = no, 1 = yes)	291 (54.1)	953 (61.0)
Diseased Vessels (0)	294 (54.6)	639 (40.9)
Diseased Vessels (1)	96 (17.8)	325 (20.8)
Diseased Vessels (2)	58 (10.8)	274 (17.5)
Diseased Vessels (3)	90 (16.7)	324 (20.7)

Age, BMI, and traffic exposure (Distance to roadway) are given as the mean (SD). Binary and categorical variables are given as the N (%). BMI = body mass index; Diseased vessels = number of diseased coronary vessels.

### Exposure assessment

Traffic-related air pollution exposure (traffic exposure) was indexed by the perpendicular distance between the patient’s residence at time of the catheterization and the nearest major roadway. Full details of the creation and use of this traffic exposure index in the CATHGEN cohort have been previously published [21] including in the context of a GWIS [24]. We defined major roadways as primary and secondary roadways. Primary roadways were major highways often distinguished by the presence of interchanges while secondary roadways were multi-lane intra and inter-city arterials. These roadways were defined by the North Carolina Department of Transportation [29], and the definition is consistent with the Master Address File/Topologically Integrated Geographic Encoding and Referencing Feature Class Code used by the U.S. Census Bureau [30]. The patient locations and North Carolina map of primary and secondary roadways were imported into ArcGIS [31] to calculate the perpendicular distance between primary residence and the nearest primary or secondary roadway. The mean distance was 0.78 km (SD = 0.71, inter-quartile range (IQR) = 0.94) in the AA subgroup and 0.94 km (SD = 0.82, IQR = 1.15) in the EA subgroup (**Table 1**).

### Genotyping

A total of 3,512 CATHGEN participants were genotyped on the Illumina HumanOmni 1-Quad_v1-0_C array and 2,100 of them resided within North Carolina. Of these 2,100 genotyped individuals 1562 were European-American and 538 were African-American. These race-stratified cohorts and the quality control procedures have been previously described [24,32]. The quality control consisted of removing gender mismatches, variants with low (<98%) call frequency, individuals with a low (<98%) call rate, and related individuals. Analyses were restricted to variants with a minor allele frequency (MAF) greater than 0.05.

### Statistical methods

For the race-stratified GWIS, to properly account for the ordinal nature of the number of diseased vessels outcome, a cumulative link model as implemented in the *ordinal* package [33] in R [34] was used. This model is equivalent to a proportional odds model. We adjusted for age, sex, BMI, hypertension, smoking, hyperlipidemia, and diabetes in all analyses. Additionally, Eigenstrat-calculated principal components [35] were used to adjust for racial substructure within each of the race-specific GWIS. As in previous analyses four principal components were used for the EA subgroup; two principal components were sufficient to adjust for racial substructure in the AA subgroup [24,32]. The number of principal components was based on the adjustment necessary to remove racial substructure in previous race-stratified, genome-wide analyses in CATHGEN [24,32]. Results from the cumulative link model are reported in terms of an odds ratio which signifies the increased risk due to incrementing the number of diseased vessels by one. For the interaction term, the traffic exposure was scaled to the inter-quartile range. All P-values represent a test of the null hypothesis that the regression coefficient for the interaction term equals 0.

A trans-ethnic meta-analysis was performed in the METAL software package [36] using a P-value-based meta-analysis accounting for effect direction, allele and weighted by sample size. As there were multiple instances of different minor alleles for the race-stratified cohorts, it was essential to properly account for the effect allele and differential sample sizes to insure consistent meta-analysis results.

Given the often low power of interaction studies [37,38], in addition to examining any genome-wide significant associations (P < 5x10^-8^) we used a suggestive cutoff of P < 1x10^-5^ in a single subgroup for the initial analyses. This suggestive cutoff has been used in previous GWIS [24,25] to determine interaction associations worthy of being examined for replication in independent cohorts. We used P < 0.05 as the cutoff for replication in our race-stratified cohorts. For the trans-ethnic meta-analysis, P < 1x10^-5^ was used to indicate suggestive association in the full dataset.

Functional relevance of each single nucleotide polymorphism (SNP) involved in a suggestive interaction with traffic exposure was assessed using multiple publically available databases. To identify variants with potential regulatory or epigenetic implications (e.g., CpG sites), the sequence surrounding each SNP was examined using the NCBI dbSNP database [39]. We identified potential regulatory intergenic variants by integrating data on open chromatin regions, defined as DNAseI hypersensitivity sites, based on data from the ENCODE project [40]. In addition, recently published results on allele-specific DNA openness [41] were used to identify variants in regions related to transcription factor binding and/or nucleosome positioning. Finally, we investigated the evidence that suggestive variants may regulate gene expression by integrating data on tissue-specific expression quantitative trait loci (eQTLs) via Genotype Tissue Expression (GTEx) database Release V6 [42,43].

## Results

The study population for this analysis consisted of 2,100 individuals, residing in North Carolina. The geographic distribution of the study cohort is given in **Fig 1** while the clinical covariates are given in **Table 1**. A total of 756,588 SNPs with a MAF > 0.05 converged in the AA model and a total of 674,795 SNPs with MAF > 0.05 a converged in the EA model. The IQR for the traffic exposure variable for the AA and EA GWIS was 0.94 km and 1.15 km respectively.

### EA results

**Fig 2:**
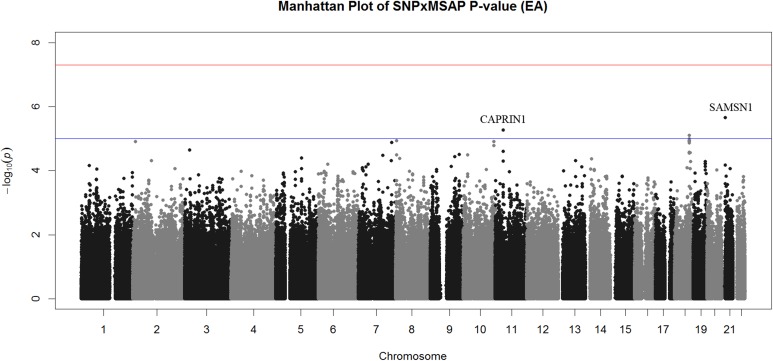
Manhattan Plot of Interaction P-values for EA GWIS. Manhattan plot of interaction P-values for the EA GWIS. Though no interactions achieved genome-wide significance (red line) interactions on chromosome 11 (*CAPRIN1*), 18, and 21 (*SAMSN1* open chromatin region) exceeded the suggestive significance threshold (blue line).

**Fig 3:**
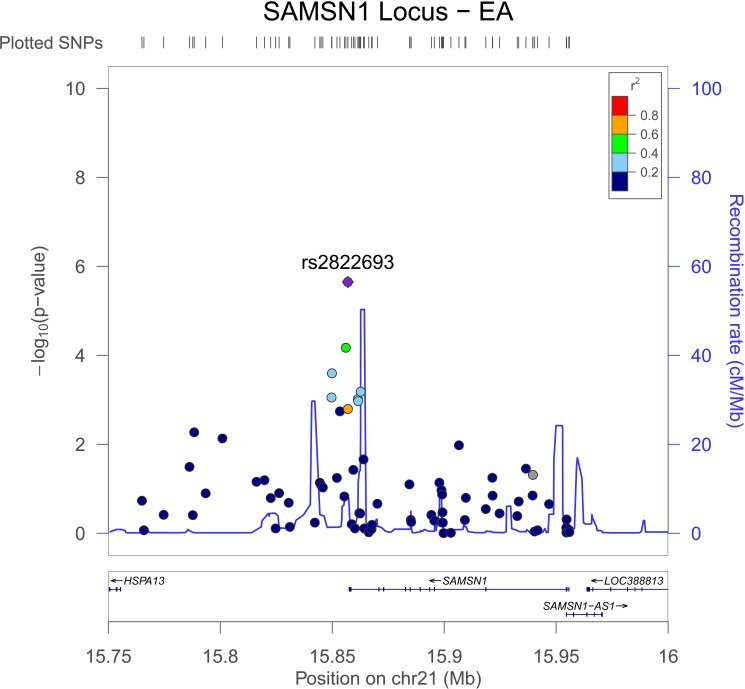
LocusZoom Plot of Open Chromatin Region Downstream of *SAMSN1* for EA GWIS. Overall the LD across the region is modest to low supporting the hypothesis that the interaction association signal is coming from rs2822693 or an untyped variant in high LD with it, likely restricted to the region between the two recombination peaks. For plotting 1000 Genomes data was used (population = EUR) in genome build hg19.

Three interactions exceeded the threshold for suggestive evidence of association (P < 1x10^-5^) (**Fig 2, Table 2A**). All of the suggestive EA interactions were in intergenic regions. The most significant association was rs2822693 (P = 2.2x10^-6^), an intergenic SNP on chromosome 21 located near *SAMSN1*. This region contained relatively few typed variants and the most significant variants were those that were in LD with rs2822693 (**Fig 3**). Rs2822693 was located in a DNAse I hypersensitivity site present in hematopoetic stem cells. *SAMSN1* expression was highest in hematopoetic stem cells and the DNAse I hypersensitivity site containing rs2822693 was highly correlated with *SAMSN1* expression (dnase.genome.duke.edu [40]). Rs12285326 was located near *CAPRIN1* on chromosome 21 however was not in a DHS site or an eQTL for this gene.

**Table 2:** GWIS interactions with P < 1x10^-5^.

**a. EA GWIS interaction P < 1x10-5**						
SNP	Locus	AA OR (CI)	AA P	EA OR	EA P	Minor Allele (AA/EA)
rs12285326	*SAMSN1* (DHS)	1.42 (0.77, 1.72)	0.11	0.61(0.32, 0.62)	5.4x10^-6^	A/A
rs2822693	*CAPRIN1* (Intergenic)	1.15 (0.92, 2.19)	0.49	0.45 (0.49, 0.76)	2.3x10^-6^	A/A
rs1108775	Intergenic	1.00 (0.72, 1.39)	0.99	1.55 (1.28, 1.87)	7.9x10^-6^	G/A
**b. AA GWIS interaction P < 1x10-5**						
SNP	Locus	AA OR (CI)	AA P	EA OR	EA P	Minor Allele (AA/EA)
rs1856746	*FCAMR* (Intron)	2.96 (1.89, 4.64)	2.3x10^-6^	0.83 (0.68, 0.99)	0.048	A/G
rs2791713	*FCAMR-PIGR* (Intergenic)	3.00 (1.90, 4.73)	2.4x10^-6^	0.80 (0.66, 0.97)	0.02	G/A
rs291096	*PIGR* (V183V)	2.97 (1.88, 4.67)	2.7x10^-6^	0.85 (0.77, 1.02)	0.09	A/G
rs17366136	Intergenic	2.47 (1.66, 3.68)	8.1x10^-6^	0.86 (0.71, 1.04)	0.12	A/A
rs11012265	Intergenic	0.46 (0.33, 0.65)	9.1x10^-6^	1.01 (0.83, 1.21)	0.95	A/A

We observed three suggestive interactions in the EA GWIS **(a)** and five suggestive interactions in the AA GWIS **(b)** two of which replicated: rs1856746 and rs2791713. Rs1108775, rs17366136 and rs11012265 were all located in intergenic regions not near any annotated gene. Genotypes were coded according to the number of copies of the minor allele. BP = base pair location; CI = 95% confidence interval; DHS = DNAse I hypersensitivity site; OR = odds ratio for interaction from cumulative link model; SNP = SNP involved in the SNP-traffic exposure interaction

### AA results

**Fig 4:**
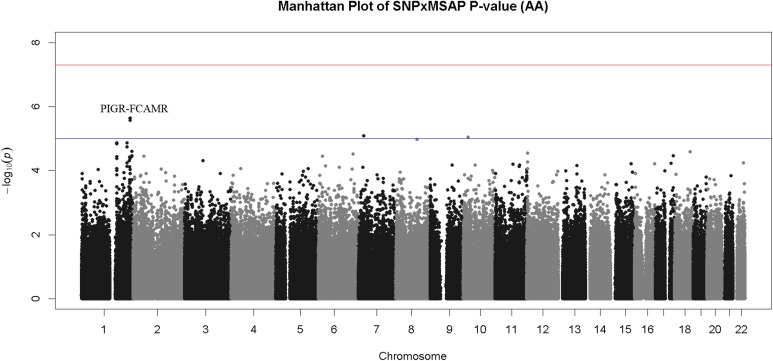
Manhattan Plot of Interaction P-values for AA GWIS. Though no interactions exceeded the genome-wide significance threshold a cluster of SNP-by-TRAP interactions on chromosome 1 (*FCAMR-PIGR* locus) exceeded the threshold for suggestive evidence of association (P < 1x10^-5^, blue line).

A total of five interactions had a P < 1x10^-5^ in the AA GWIS (**Table 2B, Fig 4**). The *FCAMR-PIGR* locus on chromosome 1 accounted for the three most significant interactions in the AA GWIS: rs1856746 (P = 2.3x10^-6^) is in an intron of *FCAMR*; rs2791713 (P = 2.4x10^-6^) is located in the intergenic region between *FCAMR* and *PIGR*; and rs291096 is a synonymous variant in *PIGR* (P = 2.7x10^-6^, V183V). Both of the remaining suggestive interactions were with intergenic variants (**Table 2B**). Rs1856746 and rs2791713 both replicated in the EA GWIS. The minor allele was flipped between the EA and AA cohorts for rs1856746 and rs791713. The coding variant rs291096 had an interaction P = 0.09 in the EA cohort, narrowly missing the threshold for replication. None of the suggestive interactions from the EA cohort replicated (**Table 2**).

### Interactions in the FCAMR-PIGR locus

**Fig 5:**
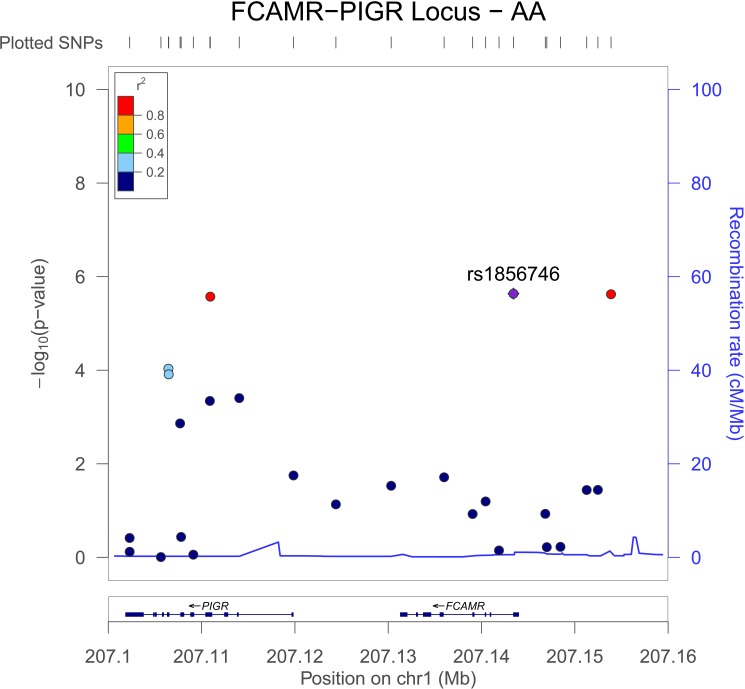
LocusZoom Plot of *FCAMR-PIGR* locus for African-Americans. LD is calculated relative to the most significant variant rs1856746. LD across the three most significant variants within the region is substantial however all other variants in the region show little correlation with rs291096 despite the relatively low recombination rate across the region. For plotting 1000 Genomes data is used (population AFR) in genome build hg19.

**Fig 6:**
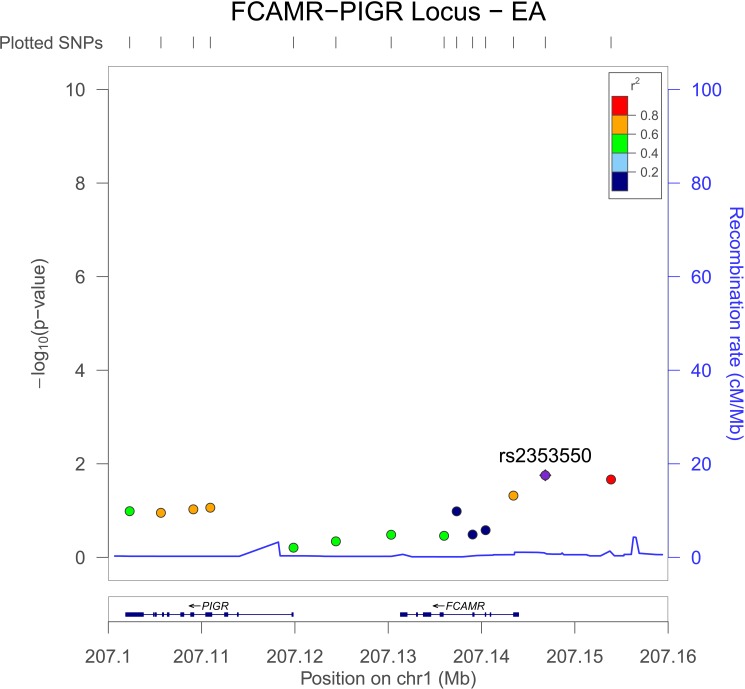
LocusZoom Plot of *FCAMR-PIGR* locus for European-Americans. Similar to what was observed in the African-Americans (**Fig 5**) substantial LD is observed in the *FCAMR-PIGR* region particularly between the replicated variant (rs185674) and variants in *PIGR*. For plotting 1000 Genomes data is used (population = EUR) in genome build hg19.

We examine all genotyped SNPs in the *FCAMR-PIGR* locus, defined as chromosome 1 base pairs 207101866–207144307 (genome build GRCh37). There were five variants in addition to rs1856746 in *FCAMR*, one of which, rs11119925, had a nominally significant interaction P in the AA and EA GWIS (AA P = 0.019, EA P = 0.034, **Table A(a) in S1 File**. There were 11 variants in *PIGR* represented in our GWIS; In addition to the synonymous variant rs291096, five additional SNPs had interactions with P < 0.05 in the AA GWIS: two were intronic variants, two synonymous variants, and one a missense variant (rs29110, A -> V, AA P = 1.2x10^-4^, **Table A(b) in S1 File**). The LD in African ancestry individuals amongst the coding variants in the *PIGR-FCAMR* locus was low (r^2^ < 0.40); however, there was substantial LD (r^2^ > 0.60) between the most significant *PIGR* and *FCAMR* variants (**Fig 5**). The observed LD for *PIGR* and *FCAMR* variants was reduced in European ancestry individuals but still substantial (**Fig 6**).

### Meta-analysis

In a sample size weighted meta-analysis combining the EA and AA cohorts, the most significant interaction was with rs10830090 on chromosome 10, located in an intron of *RARS2*, a mitochondrial arginine t-RNA synthase (**Table B in S1 File**). Mutations in *RARS2* cause a neurodegenerative disorder, pontocerebellar hypoplasia type 6 [44]. Mitochondrial function is linked to both air pollution and cardiovascular disease as ultra-fine particulates can cause mitochondrial damage [45] linked to cardiovascular disease [46]. All of the other suggestive meta-analysis interactions were intergenic.

### Integration of suggestive associations with multi-tissue eQTL data

Investigation of the suggestive AA and EA GWIS variants in data from the GTEx consortium [42] revealed that rs291096, rs1856746, and rs2791713—the three *FCAMR-PIGR* locus variants—are eQTLs for *RP11-564A8*.*8* in lymphocytes. Rs17366136 is an eQTL for *AC073072*.*5* in tibial nerve and artery tissue (**Table C in S1 File**). From the meta-analysis, rs6894083 was an eQTL for *MARCH3* in skeletal muscle; it was also an eQTL for C5orf63 in tibial nerve and aorta (**Table D in S1 File**).

## Discussion

In this trans-ethnic meta-analysis of gene-by-traffic exposure interactions, we have uncovered multiple suggestive interactions; two of which replicated in an independent cohort. This study continues the success of recent genome-wide scans for environmental interactions associated with chronic disease. Genetic variants in the bone morphogenic protein family of genes associated were found to be associated with peripheral arterial disease [24] via a gene-by-traffic exposure GWIS. A smoking exposure GWIS identified novel genetic variants associated with coronary artery calcification, including genetic variants in *WWOX* that were also associated with peripheral arterial disease in a traffic air pollution exposure GWIS [24,25]. Here we found that the three most significant interactions in the AA GWIS were all in the *FCAMR-PIGR* locus. The two most significant interactions replicated in the EA GWIS, while the third was a synonymous coding variant within *PIGR*, which narrowly missed our replication threshold. In the EA GWIS the most significant interaction was located in *SAMSN1*. Though this interaction did not replicate in the AA GWIS, it was in a DNA hypersensitivity site in hematopoetic stem cells; further, the genetic variant correlated with the expression of *SAMSN1*.

### FCAMR-PIGR associations

We observed multiple associations between variants in the *FCAMR-PIGR* locus on chromosome 1. A coding variant in *PIGR* was associated with the number of diseased coronary vessels in our interaction model (rs291096, P = 2.3x10^-6^). The association signal from this exonic variant combined with the replication of the two most significant variants from the AA GWIS—both of which were in the *FCAMR-PIGR* locus—prompting us to further investigate this locus. Given the proximity of the two genes, their similar functions, and LD structure (**Figs 5 and 6**) we cannot completely disregard that potentially both *PIGR* and *FCAMR* are involved in mediating the effects of traffic-related air pollution on coronary atherosclerosis.

The protein products of both *FCAMR* and *PIGR* are participants in immune response. *FCAMR* is a receptor for the Fc fragments of immunoglobulin, and is upregulated by *IL1A*, an inflammatory cytokine [47]. *PIGR* is a poly-Ig receptor mediating the transport of polymeric immunoglobulin molecules [48], and is primarily found in the mucosal epithelium [49]. Both *PIGR* and *FCAMR* recognize IgA and IgM [49,50], and play an important role in the immune response in mucosal cells. *FCAMR* is widely expressed in the kidney, intestine, heart, and lung while *PIGR* is primarily found in the mucosal epithelium [49]. Both *PIGR* and *FCAMR* are implicated in IgA nephropathy, a condition where IgA immune complexes are deposited in the glomerular mesangium [47,50]. All three *FCAMR-PIGR* variants from the suggestive AA GWIS interactions were associated with the expression of *RP11-564A8*.*8* in lymphocytes. Although *RP11-564A8*.*8* is a pseudogene of unknown function, these variants potentially play an important functional role in immune cells. Though *FCAMR* has yet to be directly associated with air pollution, *PIGR* plasma levels are greater in smokers than in non-smokers, confirming that plasma *PIGR* is regulated by inhaled pollutants [51]. Finally, in a recent metabolomics quantitative trait locus analysis in this dataset a variant in *PIGR* was associated with a cluster of long-chain dicarboxyl acylcarnities (LCDA) metabolites [32]. LCDA metabolites are predictive of cardiovascular disease events [27,52].

### SAMSN1

In the EA cohort, we found suggestive evidence that rs2822693 is associated with the number of diseased coronary vessels (P = 2.2x10^-6^) via an interaction with traffic exposure. Rs2822693 is located in an open chromatin region downstream of *SAMSN1*. Open chromatin regions signify nucleosome free regions of DNA, often correlated with the binding of regulatory factors. The region containing rs2822693 was open in hematopoetic cells, and the gene expression of *SAMSN1* was highly correlated with this hypersensitivity site [40].

*SAMSN1* (also known as *HACS1*) encodes a 441 amino-acid protein with SAM and SH3 domains that indicate adaptor or scaffolding functions. Expression of *SAMSN1* is detected in several tissues including the brain, lung, heart, and hematopoetic stem cells [53]. *SAMSN1* expression is up-regulated in B cell activation signaling cascades [54], as well as in the peripheral blood mononuclear cells [55] and atherosclerotic lesions of femoral arteries [56] of PAD cases.

### Meta-analysis

In a sample size weighted meta-analysis combining the EA and AA cohorts, the most significant interaction was with rs10830090, located in an intron of *RARS2*, a mitochondrial arginine t-RNA synthase. Particulate air pollution is known to cause mitochondrial damage [45] which can lead to cardiovascular disease [46], making RARS2 an interesting candidate gene for further analyses. As this was a sample size weighted meta-analysis the most significant interactions from the AA GWIS do not have suggestive associations as their P-values were often lower in GWIS of the larger EA population.

### Uniqueness, strengths and limitations

This study is the first genome-wide interaction study of air pollution and coronary atherosclerosis. Although there are other studies of gene-air pollution interactions in cardiovascular disease [23], this is the first genome-wide examination. A strength of the study design is the use of independent race-stratified cohorts to replicate interactions; this helped to overcome the limitation of our limited sample size in each of our race-stratified cohorts by allowing us to validate observed associations in an independent sample drawn from the same population. It also allows us to observe the ethnicity driven heterogeneity in any observed associations, though this should be extended by testing these associations in ethnicities beyond EA and AA. The detailed clinical phenotyping and precise quantification of the extent of coronary artery pathology is a strength not typically available in genetic epidemiologic studies. Additionally, the distance to primary roadways is a well-validated proxy for traffic air pollution exposure that has been used in several studies [15,19,21,24,57]; it is strongly correlated with air pollution in our study area [14] and allowed us to evaluate air pollution exposure on all individuals with address information. We used the number of diseased coronary vessels as a measure of coronary atherosclerosis. This measure allowed us to evaluate clinically significant atherosclerosis as assessed by a physician across multiple coronary vessels. A strength of our measure is that it allows for assessment of clinically significant atherosclerosis across multiple vessels, however a limitation is that it does not assess subclinical atherosclerosis that may still be impacted by these associations.

The primary limitation of this study is that we do not have additional replication cohorts. For some of the SNPs that achieved a suggestive level of significance we were able to replicate the association in an independent cohort of a different ethnicity drawn from the same source population, which strengthens our confidence in the associations. Of course, replication in additional independent populations is required. Until then these current results require careful interpretation. A secondary limitation is not being able to assess the specific traffic air pollution components contributing to the association and evaluate their association with air pollution sources beyond traffic. Associations with traffic exposure zones may be able to refine the associations, but would require detailed maps of land usage and traffic patterns across the state, which are currently only available for a limited number of counties [21,58]. Future studies should assess whether these genes only interact with traffic-generated air pollution or if sources such as biomass burning or wood smoke generate similar associations. In this study we are also limited to a single geographic region. To better evaluate generalizability, these associations should be validated in other regions where local traffic emissions may differ from what we observed.

## Conclusion

We use a race-stratified genome-wide interaction study design with meta-analysis to investigate the role of genetic variants in modifying the association between traffic related air pollution and coronary atherosclerosis. Using this approach, we identified several novel candidate genes that may link air pollution and coronary atherosclerosis. Candidate gene interaction studies have added much to our knowledge of air pollution and coronary atherosclerosis [23]; our findings add to these studies by implicating additional inflammatory response genes (*FCAMR* and *PIGR*). We also found an EA-specific interaction in a regulatory region associated with *SAMSN1*, a gene previously associated with vascular disease. These results demonstrate the importance of considering both genetic and environmental factors in assessments of cardiovascular disease risk while highlighting the need for further systems biology research in this field.

## Supporting information

S1 File Contains Supplemental Tables A-D.
(DOCX)
