# Supplementary material for: A genome-wide trans-ethnic interaction study links the PIGR-FCAMR locus to coronary atherosclerosis via interactions between genetic variants and residential exposure to traffic
Source: PLoS One. 2017 Mar 29;12(3):e0173880. doi: 10.1371/journal.pone.0173880 (PMC5371323; doi:10.1371/journal.pone.0173880)
Supplement: S1 File — Contains Supplemental Tables A-D. (DOCX) [file pone.0173880.s001.docx]

**Supplemental Materials**

**Table A. *FCAMR (a)* and *PIGR* (b) interactions.** All odds ratios (OR) and P-values (P) refer to the interaction term in the SNP-traffic exposure interaction model. Variants with a minor allele frequency (MAF) < 0.05 are simply have their OR, P, and MAF columns marked “MAF < 0.05” as they were not analyzed per the outlined Methods. AA = African-Americans; EA = European-Americans; MAF = minor allele frequency; OR = odds ratio; SNP = single nucleotide polymorphism

| (a) *FCAMR* |  |  |  |  |  |  |
| --- | --- | --- | --- | --- | --- | --- |
| **SNP** | **OR (AA)** | **P (AA)** | **MAF AA** | **OR (EA)** | **P (EA)** | **MAF (EA)** |
| rs1856746 | 2.96 | 2.31E-06 | 0.15 | 0.83 | 0.048 | 0.46 |
| rs11119925 | 0.66 | 0.02 | 0.36 | 1.11 | 0.34 | 0.27 |
| rs11119933 | 0.71 | 0.06 | 0.31 | 1.16 | 0.26 | 0.18 |
| rs11576146 | 0.69 | 0.12 | 0.18 | 1.14 | 0.32 | 0.18 |
| rs2000059 | MAF < 0.05 | MAF < 0.05 | MAF < 0.05 | 1.30 | 0.10 | 0.11 |
| rs17018470 | 0.93 | 0.71 | 0.20 | MAF < 0.05 | MAF < 0.05 | MAF < 0.05 |
| (b) *PIGR* |  |  |  |  |  |  |
| **SNP** | **OR (AA)** | **P (AA)** | **MAF (AA)** | **OR (EA)** | **P (EA)** | **MAF (EA)** |
| rs291096 | 2.97 | 2.67E-06 | 0.16 | 0.85 | 0.09 | 0.46 |
| rs172361 | 0.50 | 9.24E-05 | 0.37 | MAF < 0.05 | MAF < 0.05 | MAF < 0.05 |
| rs291102 | 0.50 | 1.22E-04 | 0.35 | MAF < 0.05 | MAF < 0.05 | MAF < 0.05 |
| rs291090 | 0.53 | 3.94E-04 | 0.40 | MAF < 0.05 | MAF < 0.05 | MAF < 0.05 |
| rs291097 | 0.54 | 4.52E-04 | 0.40 | MAF < 0.05 | MAF < 0.05 | MAF < 0.05 |
| rs908701 | 0.58 | 1.37E-03 | 0.44 | MAF < 0.05 | MAF < 0.05 | MAF < 0.05 |
| rs7542760 | 0.83 | 0.36 | 0.22 | MAF < 0.05 | MAF < 0.05 | MAF < 0.05 |
| rs17017942 | 0.77 | 0.38 | 0.08 | MAF < 0.05 | MAF < 0.05 | MAF < 0.05 |
| rs13190 | 0.93 | 0.76 | 0.19 | 1.17 | 0.10 | 0.44 |
| rs2275531 | 1.04 | 0.87 | 0.22 | 1.18 | 0.09 | 0.45 |
| rs908702 | 1.01 | 0.98 | 0.19 | 1.17 | 0.11 | 0.45 |

**Table B. Meta-analysis of AA and EA GWIS interactions.** Meta-analysis association with P < 1x10^-5^ for the EA and AA GWIS. AA = African-Americans; BP = base pairs (GRCh37); Chr = chromosome; Direction = direction of association after aligning to a common minor allele; EA = European-Americans; MAF = Minor Allele Frequency; OR = odds ratio; SNP = single nucleotide polymorphism

| **SNP** | **Chr** | **BP** | **P** | **Direction** | **OR (AA)** | **P (AA)** | **MAF (AA)** | **OR (EA)** | **P (EA)** | **MAF (EA)** | **Minor Allele (AA/EA)** |
| --- | --- | --- | --- | --- | --- | --- | --- | --- | --- | --- | --- |
| rs10830090 | 10 | 129273673 | 9.88E-07 | ++ | 1.35 | 0.067 | 0.39 | 1.52 | 1.25E-05 | 0.44 | A/A |
| rs4363506 | 10 | 129274503 | 1.24E-06 | -- | 1.35 | 0.067 | 0.39 | 1.52 | 1.63E-05 | 0.44 | G/G |
| rs17078595 | 18 | 65827943 | 3.09E-06 | -- | 1.83 | 0.093 | 0.07 | 2.59 | 2.72E-05 | 0.05 | C/C |
| rs6894083 | 5 | 126541479 | 3.73E-06 | -- | 0.55 | 0.000 | 0.38 | 1.39 | 4.23E-03 | 0.20 | A/G |
| rs890710 | 5 | 143046538 | 6.06E-06 | -- | 0.63 | 0.007 | 0.28 | 0.67 | 3.70E-04 | 0.28 | A/A |
| rs1035208 | 18 | 62821954 | 6.48E-06 | ++ | 0.81 | 0.171 | 0.42 | 1.52 | 1.25E-05 | 0.50 | G/A |
| rs6900057 | 6 | 88273969 | 7.28E-06 | ++ | 0.58 | 0.020 | 0.14 | 0.46 | 5.47E-04 | 0.05 | C/C |
| rs176139 | 18 | 62781618 | 9.91E-06 | -- | 1.18 | 0.283 | 0.43 | 0.65 | 1.01E-05 | 0.48 | G/A |

**Table C.** eQTL associations for suggestive GWIS variants.

| SNP | Gene | eQTL P | eQTL Effect Size | Tissue |
| --- | --- | --- | --- | --- |
| rs291096 | *RP11-564A8.8* | 4.10E-10 | 0.63 | Cells - EBV-transformed lymphocytes |
| rs1856746 | *RP11-564A8.8* | 1.90E-10 | 0.66 | Cells - EBV-transformed lymphocytes |
| rs2791713 | *RP11-564A8.8* | 4.60E-07 | 0.55 | Cells - EBV-transformed lymphocytes |
| rs17366136 | *AC073072.5* | 3.9E-06 | -0.33 | Nerve - Tibial |
| rs17366136 | *AC073072.5* | 5.3E-06 | -0.32 | Artery - Tibial |

**Table D. eQTL association for meta-analysis results**. Rs6894083 was the only eQTL from the meta-analysis associations, and was a cis-eQTL in three tissues according to lookups in the GTEx consortium database.

| SNP | Gene | eQTL P | eQTL Effect Size | Tissue |
| --- | --- | --- | --- | --- |
| rs6894083 | *MARCH3* | 1.10E-08 | 0.37 | Muscle - Skeletal |
| rs6894083 | *C5orf63* | 1.20E-05 | -0.42 | Nerve - Tibial |
| rs6894083 | *C5orf63* | 1.60E-05 | -0.41 | Artery - Aorta |
